# CT Texture Analysis for Differentiating Bronchiolar Adenoma, Adenocarcinoma *In Situ*, and Minimally Invasive Adenocarcinoma of the Lung

**DOI:** 10.3389/fonc.2021.634564

**Published:** 2021-04-26

**Authors:** Jinju Sun, Kaijun Liu, Haipeng Tong, Huan Liu, Xiaoguang Li, Yi Luo, Yang Li, Yun Yao, Rongbing Jin, Jingqin Fang, Xiao Chen

**Affiliations:** ^1^Department of Nuclear Medicine, Daping Hospital, Army Medical University, Chongqing, China; ^2^Department of Gastroenterology, Daping Hospital, Army Medical University, Chongqing, China; ^3^Department of Radiology, Daping Hospital, Army Medical University, Chongqing, China; ^4^GE Healthcare, Shanghai, China; ^5^Chongqing Clinical Research Center for Imaging and Nuclear Medicine, Chongqing, China

**Keywords:** bronchiolar adenoma, texture analysis, computed tomography, lung adenocarcinoma, ground glass nodule

## Abstract

**Purpose:** This study aimed to investigate the potential of computed tomography (CT) imaging features and texture analysis to distinguish bronchiolar adenoma (BA) from adenocarcinoma *in situ* (AIS)/minimally invasive adenocarcinoma (MIA).

**Materials and Methods:** Fifteen patients with BA, 38 patients with AIS, and 36 patients with MIA were included in this study. Clinical data and CT imaging features of the three lesions were evaluated. Texture features were extracted from the thin-section unenhanced CT images using Artificial Intelligence Kit software. Then, multivariate logistic regression analysis based on selected texture features was employed to distinguish BA from AIS/MIA. Receiver operating characteristics curves were performed to determine the diagnostic performance of the features.

**Results:** By comparison with AIS/MIA, significantly different CT imaging features of BA included nodule type, tumor size, and pseudo-cavitation sign. Among them, pseudo-cavitation sign had a moderate diagnostic value for distinguishing BA and AIS/MIA (AUC: 0.741 and 0.708, respectively). Further, a total of 396 quantitative texture features were extracted. After comparation, the top six texture features showing the most significant difference between BA and AIS or MIA were chosen. The ROC results showed that these key texture features had a high diagnostic value for differentiating BA from AIS or MIA, among which the value of a comprehensive model with six selected texture features was the highest (AUC: 0.977 or 0.976, respectively) for BA and AIS or MIA. These results indicated that texture analyses can effectively improve the efficacy of thin-section unenhanced CT for discriminating BA from AIS/MIA.

**Conclusion:** CT texture analysis can effectively improve the efficacy of thin-section unenhanced CT for discriminating BA from AIS/MIA, which has a potential clinical value and helps pathologist and clinicians to make diagnostic and therapeutic strategies.

## Introduction

Bronchiolar adenoma (BA) is a recently recognized rare benign tumor with good prognosis that corresponds to the anatomic epithelial cellular component of bronchioles (1). According to the proportion of mucous cells and ciliated cells on the luminal surface, BA is divided into proximal type and distal type. On computed tomography (CT) images, BA often presents as a peripheral irregular-shaped small solid nodule, ground-glass nodule (GGN), or subsolid GGN with a central cavity (2, 3), which could be easily misdiagnosed as adenocarcinoma *in situ* (AIS) or minimally invasive adenocarcinoma (MIA) (4). As subtypes of lung adenocarcinomas, AIS or MIA requires surgery and is not expected to recur if removed completely (5–7). However, BA, a benign tumor, does not need surgery and just needs follow-up observation. Thus, it is important to accurately differentiate BA from AIS and MIA before operation. However, conventional CT characteristics of pulmonary nodules such as tumor size, density, shape, and margin are often insufficient for evaluation.

Clinically, biopsy is used to preoperatively confirm the diagnosis when lung cancer is suspected. This process requires an invasive procedure, which has risks including bleeding, pneumothorax, and infection (8, 9). And the pathological diagnosis through biopsy is inherently prone to sampling error due to tumor heterogeneity. Moreover, with the small size and peripheral nature of BA, preoperative diagnosis with transbronchial or percutaneous biopsy could be difficult. In addition, previous studies reported that it was extremely challenging to distinguish BA from adenocarcinoma on frozen sections, even for experienced thoracic pathologists, which results in overtreatment of patients (10). Thus, it is urgent to develop non-invasive complementary approaches to accurately discriminate BA from AIS and MIA prior to operation, which could help pathologists and clinicians to make diagnostic and therapeutic strategies.

Texture analysis is a novel imaging post-processing technique used for the quantification of image grayscale distribution features, pixel interrelationships, and spectral properties of images (11–13). Compared with conventional imaging methods, texture analysis can measure tumor heterogeneity that may not be perceptible to the human eye. Recent studies have shown potential clinical value of computer-aided texture analysis in the field of oncology, primarily preoperative diagnosis, grading, assessing progression, and response to therapy of cancer patients (14–16). In particular, CT texture analysis has shown promising results in lung cancer for subsolid/solid nodules and lung masses (17–19). However, to our knowledge, no data is available concerning the application of CT texture analysis to the BA. Therefore, we aimed to identify quantitative texture features for further evaluation as non-invasive biomarkers. Such biomarkers could potentially be used to distinguish BA from AIS and MIA.

## Materials and Methods

### Patients

This study was approved by the ethics committee of Daping Hospital, and the informed consent requirement was waived. In this retrospective study, from October 2017 to June 2020, 86 patients underwent surgical resection of tumors presenting as solitary pulmonary nodule, including solid nodule, GGN, or subsolid GGN, on CT images and pathologically diagnosed as BA, AIS, or MIA. AIS and MIA were diagnosed according to the World Health Organization 2015 criteria as confirmed by surgery (20). BA was diagnosed based on morphologically identifying a continuous basal cell layer, which was followed by immunohistochemical confirmation using the basal cell markers p40, p63, and CK5/6 (1, 21, 22). Tissue specimens were reviewed by pathologists with 10 years of experience in lung pathology. The inclusion criteria were as follows: (a) thin-section CT scans were performed before surgery; (b) lesions presented as solitary pulmonary nodule on thin-section CT images; (c) biopsy, surgery, chemotherapy, and radiotherapy were not performed for lesions before CT examination; (d) there was surgical resection and histopathological confirmation as BA, AIS or MIA; (e) the interval between CT scanning and surgery was within 30 days. The exclusion criteria were as follows: (a) tumor diameter was larger than 3 cm; (b) there were severe respiratory artifacts on CT images. In total, 15 BA, 38 AIS, and 36 MIA were enrolled. The clinical data including age, gender, smoking history, and surgical extent were collected. Patient demographics are summarized in Table 1.

**Table 1:** Demographic and clinical data of the subjects.

**Variable**		**BA (*n* = 15)**	**AIS (*n* = 38)**	**MIA (*n* = 36)**	***F*-value (*t*/**χ^2^**)**	***P*-value**
Age (years)		52.27 ± 15.91	55.95 ± 8.92	58.69 ± 11.70	1.728	0.184
Sex	Male	5/15 (33.33%)	7/38 (18.42%)	11/36 (30.56%)	1.949	0.377^a^
	Female	10/15 (66.67%)	31/38 (81.58%)	25/36 (69.44%)		
Smoking	Never	1/15 (6.67%)	2/38 (5.26%)	4/36 (11.11%)	0.908	0.635^a^
	Former or current	14/15 (93.33%)	36/38 (94.74%)	32/36 (88.89%)		
	Wedge resection	2/15 (13.33%)	4/38 (10.53%)	2/36 (5.56%)	6.538	0.162^a^
Surgery	Segmentectomy	0/15 (0%)	7/38 (18.42%)	2/36 (5.56%)		
	Lobectomy	13/15 (86.67%)	27/38 (71.05%)	32/36 (88.88%)		

*Quantitative variables are expressed as mean ± standard deviation. Qualitative variables are expressed as proportion*.

a *Chi-square test for sex, smoking, and surgery. The comparison of age among three groups was performed with ANOVA. The level of significance for intergroup differences was set at P < 0.05. BA, bronchiolar adenoma; AIS, adenocarcinoma in situ; MIA, minimally invasive adenocarcinoma*.

### CT Image Acquisition and Analysis

All CT images were obtained on a 64-detector CT scanner (LightSpeed VCT, GE Healthcare) with a breath-held helical acquisition of the entire thorax. CT parameters were as follows: tube voltage = 120 kVp; tube current = 150 mAs; detector collimation = 0.5 mm × 64; pitch = 0.625; rotation time = 0.5 s; reconstruction slice thickness = 1 mm; matrix = 512 × 512; field of view = 407 mm. All CT images were analyzed by two radiologists (JF and XL, with 13 and 9 years of experience in chest radiology, respectively) independently. Both radiologists were informed of the location of each lesion but were blinded to the pathological diagnosis. Lung nodules were divided into three types, containing pure GGNs, subsolid GGNs, and solid nodules based on thin-section unenhanced CT images. A pure GGN was defined as a nodule occupied by ground-glass opacity without solid regions. A subsolid GGN was defined as a nodule that obscured underlying vascular signs and where <50% of the nodule was observed at the mediastinal window. When more than 50% of a nodule was seen at the mediastinal window, a solid nodule was defined (3). At the lung window, we assessed the tumor size, shape (round to oval or irregular), margin (smooth, lobulated, or spiculated), tumor–lung interface (clear or fuzzy), pseudo-cavitation, and distance to pleura of the lesion. The tumor size was defined as the maximum length of the lesion in any axis (23). Pseudo-cavitation presented as an oval or round area of low attenuation in lung nodules, masses, or areas of consolidation that represent spared parenchyma, normal or ectatic bronchi, or focal emphysema rather than cavitation (24). Any interobserver discordance resulted in the radiologists reevaluating the image together and reaching a consensus.

### Volume of Interest (VOI) Segmentation and Texture Feature Extraction

The lesions were delineated on the thin-section unenhanced CT images using the ITK-SNAP software (available at www.itksnap.org) at the lung window. Two experienced radiologists (JS and HT, both with 9 years of experience in imaging) blinded to the clinical outcomes were involved in region of interest (ROI) segmentation. The whole tumor volume was determined by manually drawing an ROI along the border of the tumor on each consecutive slice covering the whole lesion. Therefore, a three-dimensional VOI was finally obtained. The texture features were automatically calculated by the AK software (Artificial Intelligence Kit, GE Healthcare). A total of 396 texture features were extracted, including six types: histogram, gray-level co-occurrence matrix (GLCM), gray-level size zone matrix (GLSZM), gray-level run-length matrix (GLRLM), form factor features, and Haralick features. The extracted texture features were standardized to remove the unit limits of each feature. The histogram described the distribution of voxel intensities within the image region defined by the mask through commonly used and basic metrics. GLRLM depicts the amount of homogeneity in specific directions. GLCM and Haralick features provide information about the gray-level value distribution of pixel pairs in all directions. GLSZM is efficient for characterizing texture homogeneity, non-periodicity, or a speckle-like texture (25).

### Statistical Analysis

Statistical analyses were performed using the SPSS software (version 20.0, IBM Corp., Armonk, NY). Kolmogorov–Smirnov and Levene tests were used for the assessment of normal distribution and equal variance. With regard to the reproducibility of volumetric and texture analysis, inter-observer reliability was assessed by an intraclass correlation coefficient (ICC) test. In general, an ICC <0 indicates no agreement, 0–0.20 slight agreement, 0.21–0.40 fair agreement, 0.41–0.60 moderate agreement, 0.61–0.80 substantial agreement, and 0.81–1 almost perfect agreement. A one-way analysis of variance (ANOVA) test was applied to assess the ability of CT imaging features to differentiate between BA and AIS/MIA. The level of significance for intergroup differences was set at *P* < 0.05. *Post-hoc* tests with Bonferroni correction were performed after observing statistical differences among the three groups. *P* < 0.017 (0.05/3) was considered significant after Bonferroni correction. Feature dimension reduction was performed as follows: first, a *t*-test or Mann–Whitney *U*-test was performed; second, univariate logistic analysis was conducted, and statistically significant features (*P* < 0.05) were chosen; third, the minimal-redundancy maximal-relevance method (mRMR) was used to remove the redundant and less-relevant features. Multivariate logistic regression analyses were performed to establish a comprehensive model with the most valuable parameters to distinguish BA from AIS or MIA. The diagnostic accuracy of different CT imaging features, textural features, and comprehensive models were evaluated by a receiver operating characteristic (ROC) analysis to obtain area under curves (AUCs), sensitivity, and specificity. The MedCalc Statistical Software (version 19.3.1, MedCalc Software Ltd, Ostend, Belgium) was used to compare differences in AUCs. Two-tailed *P*-values were calculated with a 0.05 significance level.

## Results

### Patients' Clinical Characteristics

The clinical characteristics of 15 BA, 35 AIS, and 38 MIA patients are summarized in Table 1. No significant differences were found in age, sex, smoking status, and surgery among the three groups. In our study, women (66/89, 74.16%) and patients who had smoking history (82/89, 92.13%) were more common. Lobectomy was performed in the majority of the patients (72/89, 80.90%).

### Comparison of CT Imaging Features Between BA and AIS/MIA

CT features of BA, AIS, and MIA are summarized in Table 2, and examples of BA, AIS, and MIA are shown in Figure 1. BA presented as pure GGN (2/15, 13.33%), subsolid GGN (3/15, 20.00%), or solid nodule (10/15, 66.67%), among which a solid nodule was more common in this study, whereas subsolid GGN was the common nodule type in AIS and MIA. Tumor size was larger in MIA than in BA (16.11 ± 6.07 vs. 10.53 ± 4.50, *P* < 0.001), but no significant difference was found between BA and AIS (10.53 ± 4.50 vs. 11.26 ± 3.54, *P* = 0.624). Notably, a pseudo-cavitation sign was observed more frequently in BA (10/15, 66.67%), compared to AIS (7/38, 18.42%) or MIA (9/36, 25.00%) (*P* = 0.002). No statistically significant differences with respect to tumor shape (*P* = 0.216), margin (*P* = 0.358), tumor–lung interface (*P* = 0.265), and distance to pleura (*P* = 0.623) were observed among BA, AIS, and MIA. Furthermore, ROC analysis was performed to ascertain relevant CT imaging features in differentiating BA from AIS/MIA. The results showed that the pseudo-cavitation sign had a moderate diagnostic value (AUC: 0.741, sensitivity: 81.6%, specificity: 66.7%, Supplementary Table 1) for distinguishing BA and AIS, while others demonstrated no significance (*P* > 0.05). For BA and MIA, the nodule type, tumor size, and pseudo-cavitation sign had moderate diagnostic values (AUC: 0.780, 0.763, and 0.708, respectively, Supplementary Table 2). Overall, the pseudo-cavitation sign was the CT imaging feature which had a moderate diagnostic value for differentiating BA from AIS/MIA.

**Table 2:** Differences in CT findings among BA, AIS, and MIA.

**Variable**		**BA**	**AIS**	**MIA**	***F*-value (*t*/**χ^2^**)**	***P*-value**
Diameter (mm)		10.53 ± 4.50	11.26 ± 3.54	16.11 ± 6.07	11.685	<0.001^*^^#^
Nodule type	Pure GGN	2/15 (13.33%)	2/38 (5.26%)	8/36 (22.22%)	25.99	<0.001^a^^*^^$^
	Subsolid GGN	3/15 (20.00%)	27/38 (71.05%)	26/36 (72.22%)		
	Solid nodule	10/15 (66.67%)	9/38 (23.69%)	2/36 (5.56%)		
Shape	Round to oval	1/15 (6.67%)	11/38 (28.95%)	8/36 (22.22%)	3.067	0.216^a^
	Irregular	14/15 (93.33%)	27/38 (71.05%)	28/36 (77.78%)		
Margin	Smooth	9/15 (60.00%)	13/38 (34.21%)	12/36 (33.33%)	4.375	0.358^a^
	Lobulated	5/15 (33.33%)	20/38 (52.63%)	17/36 (47.22%)		
	Spiculated	1/15 (6.67%)	5/38 (13.16%)	7/36 (19.45%)		
Tumor–lung interface	Clear	9/15 (60.00%)	25/38 (65.79%)	17/36 (47.22%)	2.658	0.265^a^
	Fuzzy	6/15 (40.00%)	13/38 (34.21%)	19/36 (52.78%)		
Pseudo-cavitation	Absent	5/15 (33.33%)	31/38 (81.58%)	27/36 (75.00%)	12.624	0.002^a^^*^^$^
	Present	10/15 (66.67%)	7/38 (18.42%)	9/36 (25.00%)		
Distance to pleura		6.18 ± 7.47	8.26 ± 8.41	8.57 ± 8.26	0.475	0.623

*Quantitative variables are expressed as mean ± standard deviation. Qualitative variables are expressed as proportion*.

a *Chi-square test for nodule type, shape, margin, tumor–lung interface, and pseudo- cavitation. The comparison of tumor maximum diameter and distance to pleura among three groups was performed with ANOVA. The level of significance for intergroup differences was set at P < 0.05*.

$ *P < 0.017 (0.05/3) BA vs. AIS, with post-hoc test, Bonferroni corrected*.

* *P < 0.017 (0.05/3) BA vs. MIA, with post-hoc test, Bonferroni corrected*.

# *P < 0.017 (0.05/3) AIS vs. MIA, with post-hoc test, Bonferroni corrected. BA, bronchiolar adenoma; AIS, adenocarcinoma in situ; MIA, minimally invasive adenocarcinoma; GGN, ground-glass nodules*.

**Figure 1:**
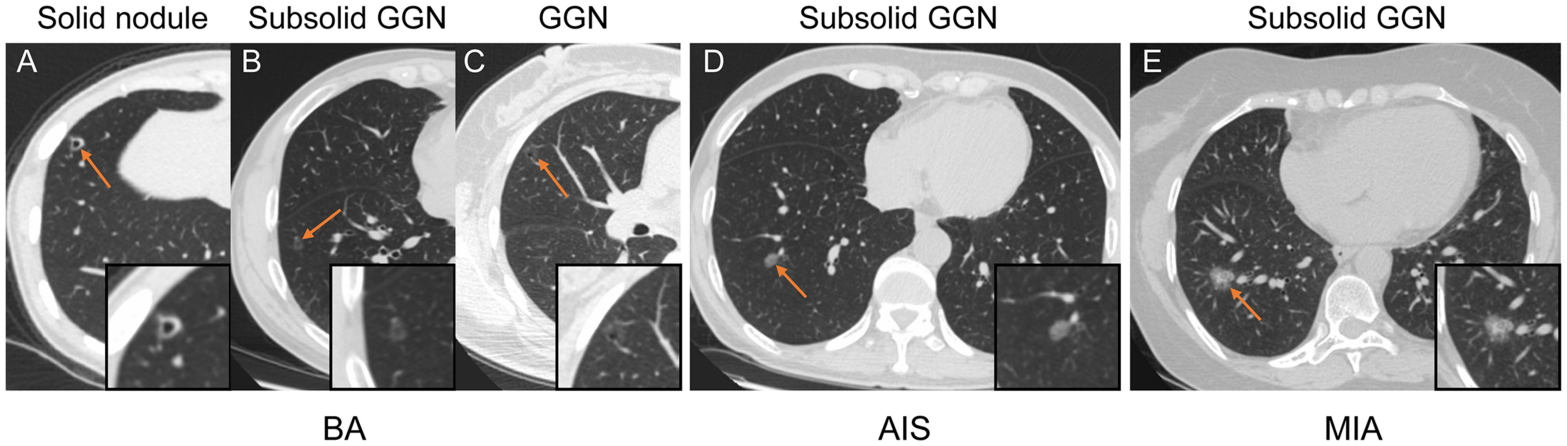
Representative CT images of patients with BA **(A–C)**, AIS **(D)**, and MIA **(E)**. **(A–C)** Axial CT image of BA presenting as a solid nodule **(A)**, subsolid GGN **(B)**, and GGN **(C)** with round to oval shape, pseudo-cavitation, smooth margins, and clear tumor–lung interface. **(D)** Axial CT image of AIS that presents as a subsolid GGN with an oval shape, smooth margins, and clear tumor–lung interface. **(E)** Axial CT image of MIA appearing as a subsolid GGN with an oval shape, small spiculated margin, shallow lobulation, and clear tumor–lung interface.

### Comparison of CT Texture Analysis Between BA and AIS/MIA

**Figure 2:**
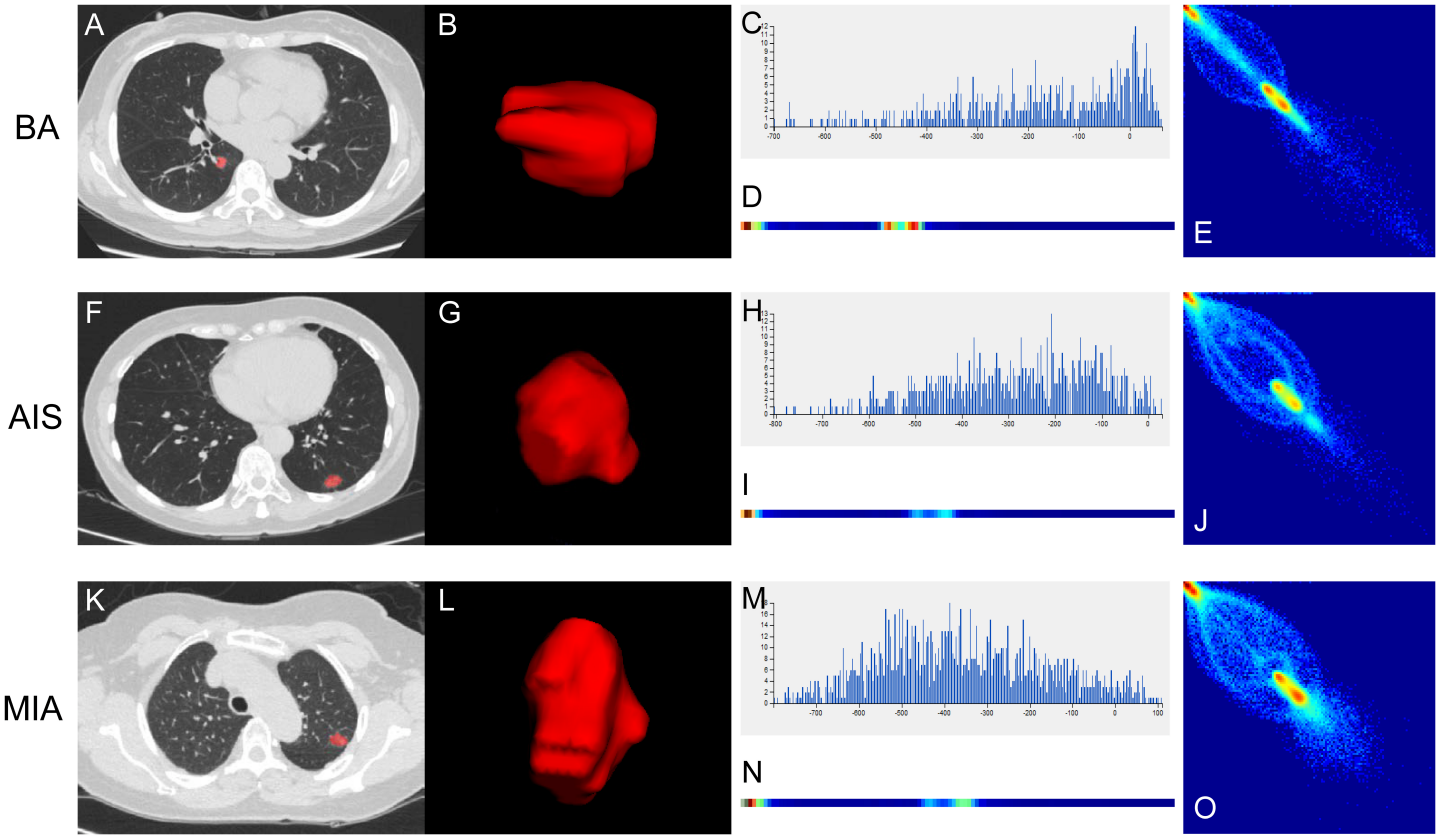
Three representative sets of CT texture features of patients with BA **(A–E)**, AIS **(F–J)**, and MIA **(K–O)**. **(A,F,K)** Thin-section unenhanced CT images. **(B,G,L)** VOIs delineated by the ITK-SNAP software. **(C,H,M)** Histograms of texture parameters of the three lesions showed a marked difference. The distribution of **(H)** and **(M)** were more dispersed than the distribution of **(C)**. **(D,I,N)** GLRLM features of the three lesions. The frequency of grayscale changes of **(D)** was more stable than that of **(I,N)**. **(E,J,O)** GLCM features of the three lesions. The distribution of **(E)** was more concentrated than the distribution of **(J,O)**.

**Figure 3:**
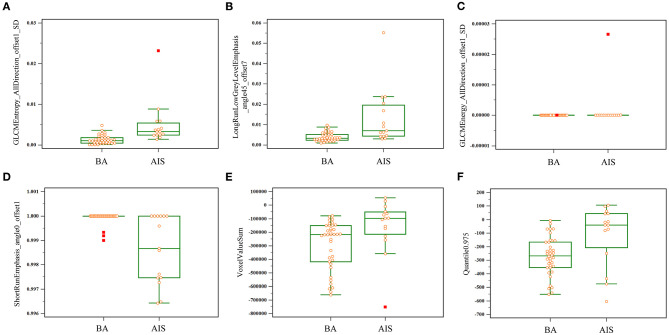
Key texture features of the most significant difference between BA and AIS. Six features, namely, GLCMEntropy_AllDirection_offset1_SD **(A)**, LongRunLowGreyLevelEmphasis_angle45_offset7 **(B)**, GLCMEnergy_AllDirection_offset1_SD **(C)**, ShortRunEmphasis_angle0_offset1 **(D)**, VoxelValueSum **(E)**, and Quantile 0.975 **(F)**, varied significantly between BA and AIS on thin-section unenhanced CT images.

**Figure 4:**
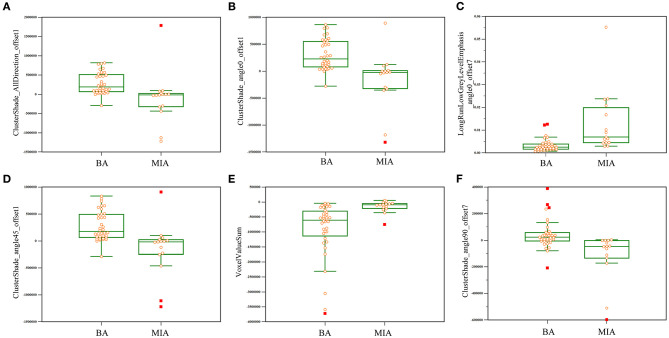
Key texture features showing the most significant difference between BA and MIA. Six features, namely, ClusterShade_AllDirection_offset1 **(A)**, ClusterShade_angle0_offset1 **(B)**, LongRunLowGreyLevelEmphasis_angle0_offset7 **(C)**, ClusterShade_angle45_offset1 **(D)**, VoxelValueSum **(E)**, and ClusterShade_angle90_offset7 **(F)**, varied significantly between BA and MIA on thin-section unenhanced CT images.

**Figure 5:**
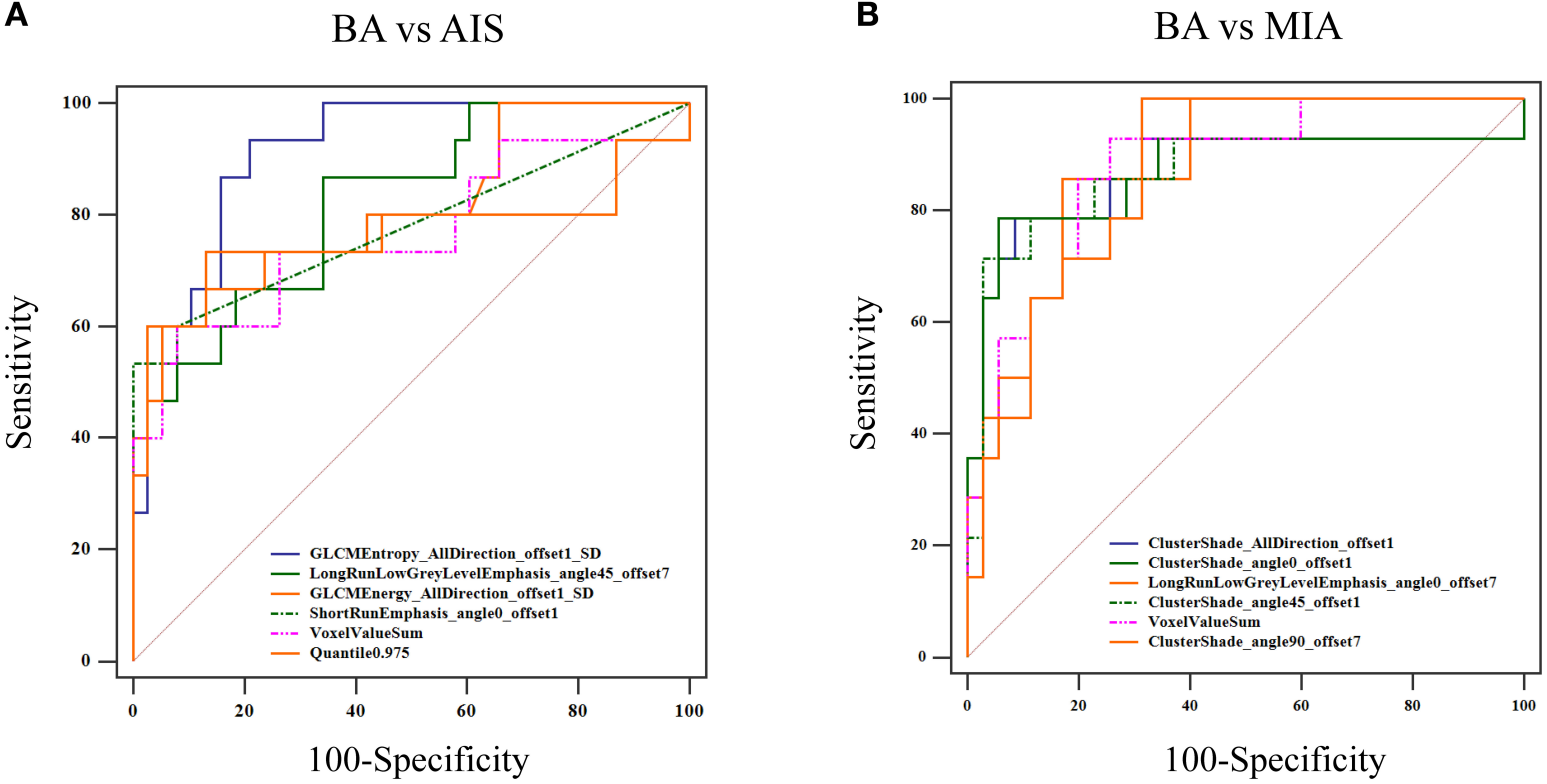
ROC curve for distinguishing BA from AIS/MIA. **(A)** For BA vs. AIS, the AUC values of GLCMEntropy_AllDirection_offset1_SD, LongRunLowGrey LevelEmphasis_angle45_offset7, GLCMEnergy_AllDirection_offset1_SD, ShortRunEmphasis_angle0_offset1, VoxelValueSum, and Quantile 0.975 were 0.912, 0.823, 0.813, 0.779, 0.763, and 0.760, respectively. **(B)** For BA vs. MIA, AUC values of ClusterShade_AllDirection_offset1, ClusterShade_angle0_offset1, LongRunLowGreyLevelEmphasis_angle0_offset7, ClusterShade_angle45_offset1, VoxelValueSum, and ClusterShade_angle90_offset7 were 0.876, 0.876, 0.874, 0.870, 0.865, and 0.857, respectively.

To improve the diagnostic value for BA and AIS/MIA, we performed the CT texture analysis of these lesions. A total of 396 texture features were extracted from unenhanced CT images. The ICC values of the inter-observer of our research were 0.82–0.98, which suggests great accordance between two readers and the reliability of VOI sketching. Three representative sets of CT texture features of patients with BA, AIS, and MIA are shown in Figure 2, which have similar CT imaging features but different characteristics of CT texture features. The histogram showed that the gray distribution of BA is more concentrated than that of AIS/MIA. The variation of GLRLM of BA is smaller than that of AIS/MIA. And the distribution of GLCM indicated that the heterogeneity of lesions in AIS/MIA was greater than that in BA. The top six texture features showing the most significant difference, namely, GLCMEntropy_AllDirection_offset1_SD (*P* < e^−4^), LongRunLowGreyLevelEmphasis_angle45_offset7 (*P* = 0.00028), GLCMEnergy_AllDirection_offset1_SD (*P* = 0.00042), ShortRunEmphasis_angle0_offset1 (*P* < e^−4^), VoxelValueSum (*P* = 0.00310), and Quantile0.975 (*P* = 0.00348), were calculated between BA and AIS (Figure 3). The diagnostic performance of each texture is shown in **Figure 5A**. Generally, an AUC > 0.9 indicates excellent diagnostic efficacy, and between 0.8 and 0.9 good diagnostic efficacy. The ROC results showed that three of these six texture features had high diagnostic values for differentiating BA from AIS (AUC > 0.8, Supplementary Table 3), among which the value of GLCMEntropy_AllDirection_offset1_SD was the highest (AUC: 0.912, sensitivity: 93.3%, specificity: 78.9%, cutoff value: 0.0018). Moreover, for BA vs. MIA, ClusterShade_AllDirection_offset1 (*P* < e^−4^), ClusterShade_angle0_offset1 (*P* < e^−4^), LongRunLowGreyLevelEmphasis_angle0_offset7 (*P* < e^−4^), ClusterShade_angle45_offset1 (*P* < e^−4^), VoxelValueSum (*P* < e^−4^), and ClusterShade_angle90_offset7 (*P* < e^−4^) were the top six texture features showing the most significant difference (Figure 4). The ROC results showed that all these six texture features had high diagnostic values for differentiating BA from MIA (AUC > 0.85, Supplementary Table 4 and Figure 5B), among which the value of ClusterShade_AllDirection_offset1 was the highest (AUC: 0.876, sensitivity: 80.0%, specificity: 91.7%, cutoff value: 28,505.6). Notably, among these features, VoxelValueSum was the texture feature showing the most significant difference in common for BA vs. AIS and BA vs. MIA. This feature was significantly greater in AIS/MIA patients than in BA patients, which had a high diagnostic value for differentiating BA from AIS/MIA simultaneously (Supplementary Tables 3, 4). Furthermore, there was no significant difference between the AUC of pseudo-cavitation and the maximum AUC of texture feature for distinguishing BA from AIS (*Z* = 1.885, *P* = 0.0595) or MIA (*Z* = 1.628, *P* = 0.1035). However, a pseudo-cavitation sign had a moderate diagnostic value for distinguishing BA and AIS (AUC: 0.741) or MIA (AUC: 0.708), while texture features had a high diagnostic value for differentiating BA from AIS (highest AUC: 0.912) or MIA (highest AUC: 0.876). These results indicate that the diagnostic values of texture features are higher than those of CT imaging features for differentiating BA from AIS/MIA.

### Model Development and Analysis

**Figure 6:**
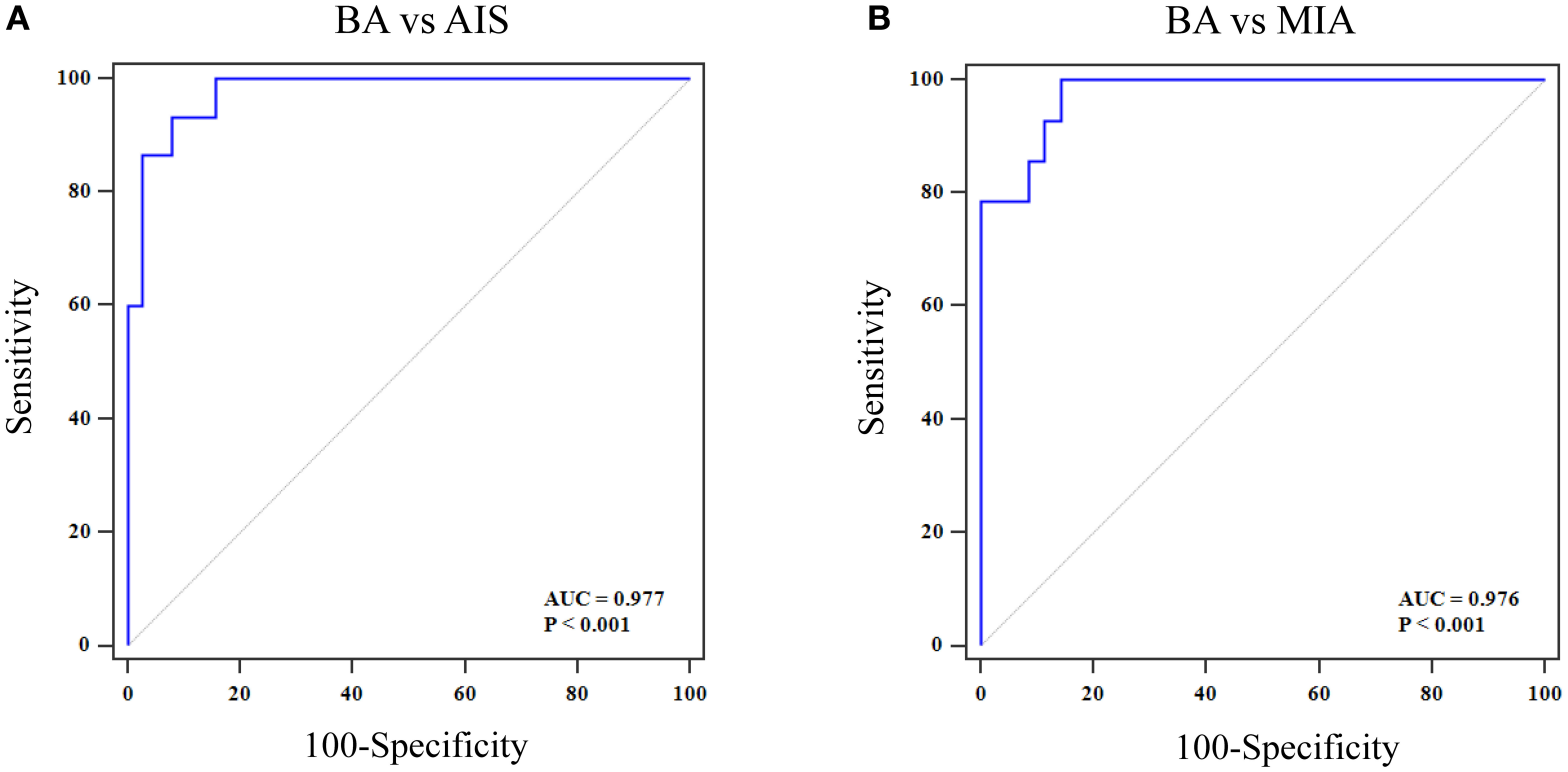
ROC curve of a comprehensive model with six selected texture features for distinguishing BA from AIS/MIA. **(A)** For BA vs. AIS, the AUC value, specificity, and sensitivity were 0.997, 92.1, and 93.3%, respectively. **(B)** For BA vs. MIA, the AUC value, specificity, and sensitivity were 0.976, 85.7, and 99.9%, respectively.

Multivariate logistic regression analyses were performed to establish a comprehensive model with six selected texture features to distinguish BA from AIS or MIA. The sensitivity, specificity, and AUC for differentiating BA from AIS were 93.3, 92.1, and 0.977 (95% CI 0.893–0.999), respectively (Figure 6A). While the sensitivity, specificity, and AUC for differentiating BA from MIA were 99.9, 85.7, and 0.976 (95% CI 0.885–0.999), respectively (Figure 6B). Further, the performance of the comprehensive model for distinguishing BA and AIS or MIA was significantly better than that of the pseudo-cavitation sign (BA vs. AIS: *Z* = 3.153, *P* = 0.0016; BA vs. MIA: *Z* = 3.508, *P* = 0.0005).

## Discussion

The present study is the first to distinguish BA from AIS/MIA using texture analysis on thin-section unenhanced CT images. In our study, we evaluated the role of CT imaging and texture analysis in differentiating BA from AIS/MIA. Pseudo-cavitation, one of the CT imaging features, could help differentiate BA from AIS and MIA. Key texture features showing the most significant difference between BA and AIS or MIA have a better distinguishing effect on disease than CT imaging features. Our data indicate that CT texture analysis demonstrates great potential in differentiating BA from AIS/MIA pre-operation.

BA, a rare benign tumor appearing as a peripherally solitary small lung nodule, was first reported in 2018 by Chang et al. (1). Although studies of BA have made new progress, some difficulties still exist in its differential diagnosis. Frozen-section diagnosis is especially challenging for BA. Invasive adenocarcinoma and BA are easily misdiagnosed based on frozen sections owing to their irregular adenoid structures and widened stroma (21, 26). Misdiagnosis of BA as invasive adenocarcinoma may lead to unnecessary interventional procedures, which leads to overtreatment of patients with BA (10). A previous study reported some CT imaging features, including pseudo-cavitation and tumor–lung interface, could help differentiate BA from AIS/MIA (2). In our study, the nodule type, tumor size, and pseudo-cavitation sign showed significant differences between BA and AIS/MIA. BA mainly manifested as a solid nodule type (66.67%), which was consistent with previous studies (3, 27–29). The pseudo-cavitation sign, which is a CT imaging feature of BA, can also be seen in adenocarcinoma, bronchioalveolar carcinoma, and infectious pneumonia (24). However, the pseudo-cavitation sign was reported to be more frequently observed in BA than in AIS and MIA (2). In our cohort, the pseudo-cavitation sign was found in 66.67% of BA and 21.62% of AIS/MIA, similar to what has been reported in a previous study. However, no significant differences were found in tumor–lung interface and tumor shape between BA and AIS/MIA, which was inconsistent with the findings of Cao et al. (2). This inconsistency may be explained by the small sample size because of the low morbidity of BA. In future, a multicenter study with a larger sample size is needed. Furthermore, we evaluated the value of nodule type, tumor size, and pseudo-cavitation sign in differentiating BA from AIS and MIA. The ROC results indicated that the pseudo-cavitation sign had a moderate diagnostic value to differentiate BA from AIS and MIA. Thus, it is necessary to develop non-invasive complementary approaches to improve the diagnostic accuracy for BA before surgery, since the diagnostic value of CT imaging features were not sufficient.

Texture analysis is an emerging imaging-based post-processing method that allows for quantification of tissue heterogeneity (30). There has been a surge in recent years in the research application of CT texture analysis in tumor identification, staging, and therapy response assessment (14–16). However, no studies have demonstrated the value of CT texture analysis in differentiating BA from AIS/MIA. Our data show that GLCMEntropy_AllDirection_offset1_SD, LongRunLowGreyLevelEmphasis_angle45_offset7, GLCMEnergy_AllDirection_offset1_SD, ShortRunEmphasis_angle0_offset1, VoxelValueSum, and Quantile0.975 were the features showing the most significant difference between BA and AIS. Meanwhile, ClusterShade_AllDirection_offset1, ClusterShade_angle0_offset1, LongRunLowGreyLevelEmphasis_angle0_offset7, ClusterShade_angle45_offset1, VoxelValueSum, and ClusterShade_angle90_offset7 were the features showing the most significant difference between BA and MIA. Moreover, to find non-invasive imaging biomarkers for detecting BA patients, we evaluated the discriminative ability of these texture features. Three of these six features had high diagnostic values in discriminating BA from AIS by performing ROC analysis independently, among which the value of GLCMEntropy_AllDirection_offset1_SD was the highest with an AUC of 0.91, sensitivity of 93.3%, and specificity of 78.9%. Meanwhile, for BA vs. MIA, the six obtained features also had high diagnostic values in discriminating BA from MIA, among which the value of ClusterShade_AllDirection_offset1 was the highest with an AUC of 0.88, sensitivity of 80.0%, and specificity of 91.7%. All these AUCs of texture features were higher than those of CT imaging features. Then, we established a comprehensive model with six selected texture features and studied the diagnostic value of the model for distinguishing BA from AIS or MIA by ROC curve analyses. The comprehensive model presented the best diagnostic value, with a significant difference relative to the pseudo-cavitation sign. Moreover, VoxelValueSum was the feature which could well-distinguish BA from AIS and MIA, simultaneously. Therefore, texture analyses can effectively improve the efficacy of thin-section unenhanced CT in discriminating BA from AIS/MIA.

There are some limitations in our study. First, this is a single-institution retrospective analysis, and the sample size is rather small because of the low morbidity of BA. Second, potential selection biases cannot be excluded since this is a retrospective study. Third, manual segmentation of GGN ROIs has a higher risk of observer bias compared to delineation with semi-automatic regression. However, the ICC values of the inter-observer of this study were 0.82–0.98, suggesting great accordance between two readers and the reliability of VOI sketching. Therefore, a multicenter program to include more BA patients may be needed, and a validation to confirm the potential value of CT texture analyses in discriminating BA from AIS/MIA may also be needed in the future.

In conclusion, our study indicated that CT texture analysis can effectively improve the efficacy of thin-section unenhanced CT for discriminating BA from AIS/MIA, which has a potential clinical value and helps pathologist and clinicians to make diagnostic and therapeutic strategies.

## Data Availability Statement

The original contributions presented in the study are included in the article/Supplementary Material, further inquiries can be directed to the corresponding author/s.

## Ethics Statement

The studies involving human participants were reviewed and approved by Research Ethics Committee of Daping Hospital, Army Medical University, Chongqing, China. The ethics committee waived the requirement of written informed consent for participation.

## Author Contributions

XC and JF conceived and designed the study. JS and YLu collected the CT and clinical data. JF and XL analyzed the CT data. HT and JS extracted the CT texture features. HL analyzed the CT texture data. KL performed the statistics. YLi and YY prepared the figures and tables. XC and KL wrote the manuscript. JF and RJ edited the manuscript. All authors revised the manuscript and read and approved the submitted version.

## Conflict of Interest

HL was employed by company GE Healthcare. The remaining authors declare that the research was conducted in the absence of any commercial or financial relationships that could be construed as a potential conflict of interest.

## References

[1] Chang JC Montecalvo J Borsu L Lu S Larsen BT Wallace WD. Bronchiolar adenoma: expansion of the concept of ciliated muconodular papillary tumors with proposal for revised terminology based on morphologic, immunophenotypic, and genomic analysis of 25 cases. Am J Surg Pathol. (2018) 42:1010–26. 10.1097/PAS.0000000000001086 29846186 PMC8063713

[2] Cao L Wang Z Gong T Wang J Liu J Jin L. Discriminating between bronchiolar adenoma, adenocarcinoma *in situ* and minimally invasive adenocarcinoma of the lung with CT. Diagn Interv Imaging. (2020) 101:831–7. 10.1016/j.diii.2020.05.005 32482582

[3] Onishi Y Kusumoto M Motoi N Watanabe H Watanabe SI. Ciliated muconodular papillary tumor of the lung: thin-section CT findings of 16 cases. AJR Am J Roentgenol. (2020) 214:761–5. 10.2214/AJR.19.21945 31967497

[4] Si MJ Tao XF Du GY Cai LL Han HX Liang XZ. Thin-section computed tomography-histopathologic comparisons of pulmonary focal interstitial fibrosis, atypical adenomatous hyperplasia, adenocarcinoma *in situ*, and minimally invasive adenocarcinoma with pure ground-glass opacity. Eur J Radiol. (2016) 85:1708–15. 10.1016/j.ejrad.2016.07.012 27666606

[5] Kadota K Villena-Vargas J Yoshizawa A Motoi N Sima CS Riely GJ. Prognostic significance of adenocarcinoma *in situ*, minimally invasive adenocarcinoma, and nonmucinous lepidic predominant invasive adenocarcinoma of the lung in patients with stage I disease. Am J Surg Pathol. (2014) 38:448–60. 10.1097/PAS.0000000000000134 24472852 PMC4164170

[6] Gu J Lu C Guo J Chen L Chu Y Ji Y. Prognostic significance of the IASLC/ATS/ERS classification in Chinese patients-A single institution retrospective study of 292 lung adenocarcinoma. J Surg Oncol. (2013) 107:474–80. 10.1002/jso.23259 22952152

[7] Tsuta K Kawago M Inoue E Yoshida A Takahashi F Sakurai H. The utility of the proposed IASLC/ATS/ERS lung adenocarcinoma subtypes for disease prognosis and correlation of driver gene alterations. Lung Cancer. (2013) 81:371–6. 10.1016/j.lungcan.2013.06.012 23891509

[8] Merritt RE Shrager JB. Indications for surgery in patients with localized pulmonary infection. Thorac Surg Clin. (2012) 22:325–32. 10.1016/j.thorsurg.2012.05.005 22789596

[9] Ozeki N Iwano S Taniguchi T Kawaguchi K Fukui T Ishiguro F. Therapeutic surgery without a definitive diagnosis can be an option in selected patients with suspected lung cancer. Interact Cardiovasc Thorac Surg. (2014) 19:830–7. 10.1093/icvts/ivu233 25038121

[10] Guo Y Shi Y Tong J. Bronchiolar adenoma: a challenging diagnosis based on frozen sections. Pathol Int. (2020) 70:186–8. 10.1111/pin.12901 31994796

[11] Sharma N Ray AK Sharma S Shukla KK Pradhan S Aggarwal LM. Segmentation and classification of medical images using texture-primitive features: application of BAM-type artificial neural network. J Med Phys. (2008) 33:119–26. 10.4103/0971-6203.42763 19893702 PMC2772042

[12] Tourassi GD. Journey toward computer-aided diagnosis: role of image texture analysis. Radiology. (1999) 213:317–20. 10.1148/radiology.213.2.r99nv49317 10551208

[13] Gillies RJ Kinahan PE Hricak H. Radiomics: images are more than pictures, they are data. Radiology. (2016) 278:563–77. 10.1148/radiol.2015151169 26579733 PMC4734157

[14] Ganeshan B Goh V Mandeville HC Ng QS Hoskin PJ Miles KA. Non-small cell lung cancer: histopathologic correlates for texture parameters at CT. Radiology. (2013) 266:326–36. 10.1148/radiol.12112428 23169792

[15] Xie T Chen X Fang J Kang H Xue W Tong H. Textural features of dynamic contrast-enhanced MRI derived model-free and model-based parameter maps in glioma grading. J Magn Reson Imaging. (2018) 47:1099–111. 10.1002/jmri.25835 28845594

[16] Shu Z Fang S Ye Q Mao D Cao H Pang P. Prediction of efficacy of neoadjuvant chemoradiotherapy for rectal cancer: the value of texture analysis of magnetic resonance images. Abdom Radiol. (2019) 44:3775–84. 10.1007/s00261-019-01971-y 30852633

[17] Chen BT Chen Z Ye N Mambetsariev I Fricke J Daniel E. Differentiating peripherally-located small cell lung cancer from non-small cell lung cancer using a CT radiomic approach. Front Oncol. (2020) 10:593. 10.3389/fonc.2020.00593 32391274 PMC7188953

[18] Gao C Xiang P Ye J Pang P Wang S Xu M. Can texture features improve the differentiation of infiltrative lung adenocarcinoma appearing as ground glass nodules in contrast-enhanced CT? Eur J Radiol. (2019) 117:126–31. 10.1016/j.ejrad.2019.06.010 31307637

[19] Gao N Tian S Li X Huang J Wang J Chen S. Three-dimensional texture feature analysis of pulmonary nodules in CT images: lung cancer predictive models based on support vector machine classifier. J Digit Imaging. (2020) 33:414–22. 10.1007/s10278-019-00238-8 31529236 PMC7165221

[20] Micke P Mattsson JS Djureinovic D Nodin B Jirstrom K Tran L. The impact of the fourth edition of the WHO classification of lung tumours on histological classification of resected pulmonary NSCCs. J Thorac Oncol. (2016) 11:862–72. 10.1016/j.jtho.2016.01.020 26872818

[21] Kamata T Yoshida A Kosuge T Watanabe S Asamura H Tsuta K. Ciliated muconodular papillary tumors of the lung: a clinicopathologic analysis of 10 cases. Am J Surg Pathol. (2015) 39:753–60. 10.1097/PAS.0000000000000414 25803171

[22] Chuang HW Liao JB Chang HC Wang JS Lin SL Hsieh PP. Ciliated muconodular papillary tumor of the lung: a newly defined peripheral pulmonary tumor with conspicuous mucin pool mimicking colloid adenocarcinoma: a case report and review of literature. Pathol Int. (2014) 64:352–7. 10.1111/pin.12179 25047506

[23] Bankier AA MacMahon H Goo JM Rubin GD Schaefer-Prokop CM Naidich DP. Recommendations for measuring pulmonary nodules at CT: a statement from the fleischner society. Radiology. (2017) 285:584–600. 10.1148/radiol.2017162894 28650738

[24] Hansell DM Bankier AA MacMahon H McLoud TC Muller NL Remy J. Fleischner society: glossary of terms for thoracic imaging. Radiology. (2008) 246:697–722. 10.1148/radiol.2462070712 18195376

[25] Zhang Y Yu S Zhang L Kang L. Radiomics based on CECT in differentiating kimura disease from lymph node metastases in head and neck: a non-invasive and reliable method. Front Oncol. (2020) 10:1121. 10.3389/fonc.2020.01121 32850321 PMC7397819

[26] Travis WD Brambilla E Noguchi M Nicholson AG Geisinger K Yatabe Y. Diagnosis of lung adenocarcinoma in resected specimens: implications of the 2011 international association for the study of lung cancer/American thoracic society/European respiratory society classification. Arch Pathol Lab Med. (2013) 137:685–705. 10.5858/arpa.2012-0264-RA 22913371

[27] Abe M Osoegawa A Miyawaki M Noda D Karashima T Takumi Y. Ciliated muconodular papillary tumor of the lung: a case report and literature review. Gen Thorac Cardiovasc Surg. (2020) 68:1344–9. 10.1007/s11748-019-01252-x 31749068

[28] Onishi Y Ito K Motoi N Morita T Watanabe SI Kusumoto M. Ciliated muconodular papillary tumor of the lung: 18F-FDG PET/CT findings of 15 cases. Ann Nucl Med. (2020) 34:448–52. 10.1007/s12149-020-01457-8 32172513

[29] Shao K Wang Y Xue Q Mu J Gao Y Wang Y. Clinicopathological features and prognosis of ciliated muconodular papillary tumor. J Cardiothorac Surg. (2019) 14:143. 10.1186/s13019-019-0962-3 31340823 PMC6651997

[30] Cannella R Borhani AA Minervini MI Tsung A Furlan A. Evaluation of texture analysis for the differential diagnosis of focal nodular hyperplasia from hepatocellular adenoma on contrast-enhanced CT images. Abdom Radiol. (2019) 44:1323–30. 10.1007/s00261-018-1788-5 30267107

