# Supplementary material for: CT Texture Analysis for Differentiating Bronchiolar Adenoma, Adenocarcinoma In Situ, and Minimally Invasive Adenocarcinoma of the Lung
Source: Front Oncol. 2021 Apr 26;11:634564. doi: 10.3389/fonc.2021.634564 (PMC8109050; doi:10.3389/fonc.2021.634564)
Supplement: Supplementary file 1 [file Data_Sheet_1.docx]

Supplementary Material

Table S1. CT imaging features of disease identification performance for BA vs AIS.

| CT imaging feature | AUC | 95% CI | SEN | SPE | *P* value |
| --- | --- | --- | --- | --- | --- |
| Pseudo-cavitation | 0.741 | 0.603-0.852 | 0.816 | 0.667 | 0.001 |
| Nodule type | — | — | — | — | 0.054 |
| Tumor size | — | — | — | — | 0.615 |

Notes: AUC, areas under the curves; 95% CI, 95% confidence interval (binomial exact); SEN, sensitivity; SPE, specificity.

Table S2. CT imaging features of disease identification performance for BA vs MIA.

| CT imaging feature | AUC | 95% CI | SEN | SPE | *P* value |
| --- | --- | --- | --- | --- | --- |
| Pseudo-cavitation | 0.708 | 0.564-0.827 | 0.750 | 0.667 | 0.004 |
| Nodule type | 0.780 | 0.642-0.884 | 0.944 | 0.667 | 0.001 |
| Tumor size | 0.763 | 0.623-0.871 | 0.778 | 0.800 | 0.001 |

Notes: AUC, areas under the curves; 95% CI, 95% confidence interval (binomial exact); SEN, sensitivity; SPE, specificity.

Table S3. Six texture features of disease identification performance for BA vs AIS.

| Texture feature | AUC | 95% CI | SEN | SPE | Cutoff |
| --- | --- | --- | --- | --- | --- |
| GLCMEntropy_AllDirection_offset1_SD | 0.912 | 0.802-0.972 | 0.933 | 0.789 | ＞0.0018 |
| LongRunLowGreyLevelEmphasis_angle45_offset7 | 0.823 | 0.693-0.914 | 0.867 | 0.658 | ＞0.00357 |
| GLCMEnergy_AllDirection_offset1_SD | 0.813 | 0.682-0.907 | 0.600 | 0.974 | ＞10^-8^ |
| ShortRunEmphasis_angle0_offset1 | 0.779 | 0.644-0.881 | 0.533 | 0.999 | ≤0.99867 |
| VoxelValueSum | 0.763 | 0.626-0.869 | 0.600 | 0.921 | ＞-109340 |
| Quantile0.975 | 0.760 | 0.623-0.866 | 0.733 | 0.868 | ＞-96.9145 |

Notes: AUC, areas under the curves; 95% CI, 95% confidence interval (binomial exact); SEN, sensitivity; SPE, specificity.

| Texture feature | AUC | 95% CI | SEN | SPE | Cutoff |
| --- | --- | --- | --- | --- | --- |
| ClusterShade_AllDirection_offset1 | 0.876 | 0.742-0.949 | 0.800 | 0.917 | ≤28505.6 |
| ClusterShade_angle0_offset1 | 0.876 | 0.740-0.947 | 0.800 | 0.945 | ≤11802.5 |
| LongRunLowGreyLevelEmphasis_angle0_offset7 | 0.874 | 0.757-0.956 | 0.867 | 0.806 | ＞0.0043 |
| ClusterShade_angle45_offset1 | 0.870 | 0.735-0.945 | 0.800 | 0.889 | ≤27825.7 |
| VoxelValueSum | 0.865 | 0.750-0.952 | 0.933 | 0.722 | ＞-394145 |
| ClusterShade_angle90_offset7 | 0.857 | 0.740-0.947 | 1.000 | 0.667 | ≤-3255.26 |

Table S4. Six texture features of disease identification performance for BA vs MIA.

Notes: AUC, areas under the curves; 95% CI, 95% confidence interval (binomial exact); SEN, sensitivity; SPE, specificity.
